# Association of Sublingual Varices With Heart- and Cerebrovascular Disease

**DOI:** 10.1016/j.identj.2023.08.003

**Published:** 2023-09-22

**Authors:** Håkan Bergh, Margit Albrektson, Clovis Kastberg, Lennart Hedström

**Affiliations:** a School of Public Health and Community Medicine, Institute of Medicine, University of Gothenburg, Gothenburg, Sweden; b Department of Research & Development Unit, Hospital Varberg, Region Halland, Halmstad Sweden; c Public Dental Services, Västra Vall, Varberg, Sweden; d Tre Tandläkare, Varberg, Sweden

**Keywords:** Cardiovascular diseases, Ischaemic heart disease, Cerebrovascular diseases, Varicose veins

## Abstract

**Objective:**

The aim of this research was to investigate whether sublingual varices (SV) are associated with new events of ischaemic heart disease (IHD) and new events of cerebrovascular disease.

**Methods:**

A prospective observational study was conducted amongst 1139 dental patients aged between 48 and 84 years across 2 cohorts (201 enrolled from 2010–2013 and 938 from 2018–2020). Participants provided baseline data on demographics, risk factors, and medical diagnoses, followed by an assessment of their tongue's ventral surface to classify veins as either having no sublingual varices (nSV) or having sublingual varices (SV). Information regarding medical diagnoses was gathered during the follow-up period from both participants and their medical records. The primary outcome variables were the onset of new IHD and new cerebrovascular disease events. Comparisons were made between participants with SV and nSV.

**Results:**

The study population comprised 54% women with an average age of 66 years. SV were present in 33% of participants. Those with SV predominantly were male, older, and smokers; had a higher body mass index, and exhibited more instances of hypertension, diabetes, and dyslipidaemia than those with nSV. New occurrences of IHD (4.5% vs 1.8%, *P* = .009) and cerebrovascular disease (4.2% vs 2.0%, *P* = .026) were more prevalent in the SV group compared with the nSV group. The link between SV and new IHD events persisted even after adjustments for sex, age, and smoking habits (OR, 2.26; 95% CI, 1.07—4.76), but not for new cerebrovascular disease events (OR, 1.77; 95% CI, 0.84—3.71).

**Conclusions:**

Our study identifies a correlation between SV and new events of IHD, but not new events of cerebrovascular disease. The detection of SV occurred prior to the IHD events, suggesting a temporal relationship. This novel finding proposes a potential shared pathophysiology between IHD and SV.

## Introduction

Oral varicosities (OV), enlarged veins in the oral cavity, can manifest in various locations including the buccal mucosa, interior of the lips, labial commissures, palate, floor of the mouth, and particularly the ventral surface of the tongue.^1^ OV correlates with age, family history, medical conditions, and other risk factors.^2^ The most common place of OV (76.2%)^3^ is the ventral surface of the tongue which, due to the location, can be named sublingual varices (SV).

The veins on the undersurface of the tongue can change and become dilated; this condition is called SV. This condition is easy to see visually during a regular examination of the oral cavity. The prevalence of SV is approximately 30% depending on the age of the study group, and SV are more common in old age.^4^, ^5^, ^6^, ^7^ The pathogenesis of SV is unknown, and they have long been regarded as an insignificant ageing phenomenon. The association of SV and OV with lower limb varices has demonstrated both positive and negative relationships in different studies.^2^^,^^8^^,^^9^ After a connection was found between SV and smoking^4^, ^5^, ^6^ and with hypertension,^5^^,^^7^^,^^10^ scientific interest in the phenomenon has increased.

Some studies have suggested an association between SV and cardiovascular disease (CVD),^4^^,^^6^^,^^7^ defined according to the International Classification of Diseases (ICD-10),^11^ including hypertension, ischaemic heart disease (IHD; myocardial infarction, angina pectoris), atrial fibrillation, valvular heart disease, and stroke. The aforementioned studies, mainly cross-sectional, demonstrate that SV often coexists with CVD.

IHD and stroke share risk factors such as smoking, high blood pressure, diabetes, abdominal obesity, and dyslipidaemia, all of which have demonstrated links with SV.^2^^,^^4^, ^5^, ^6^, ^7^^,^^9^^,^^10^

Despite prior studies affirming the coexistence of SV and CVD, the nature of their relationship remains underexplored. The aim of this study was to investigate whether sublingual varices are associated with new events of IHD and new events of cerebrovascular disease.

## Methods

**Fig. 1:**
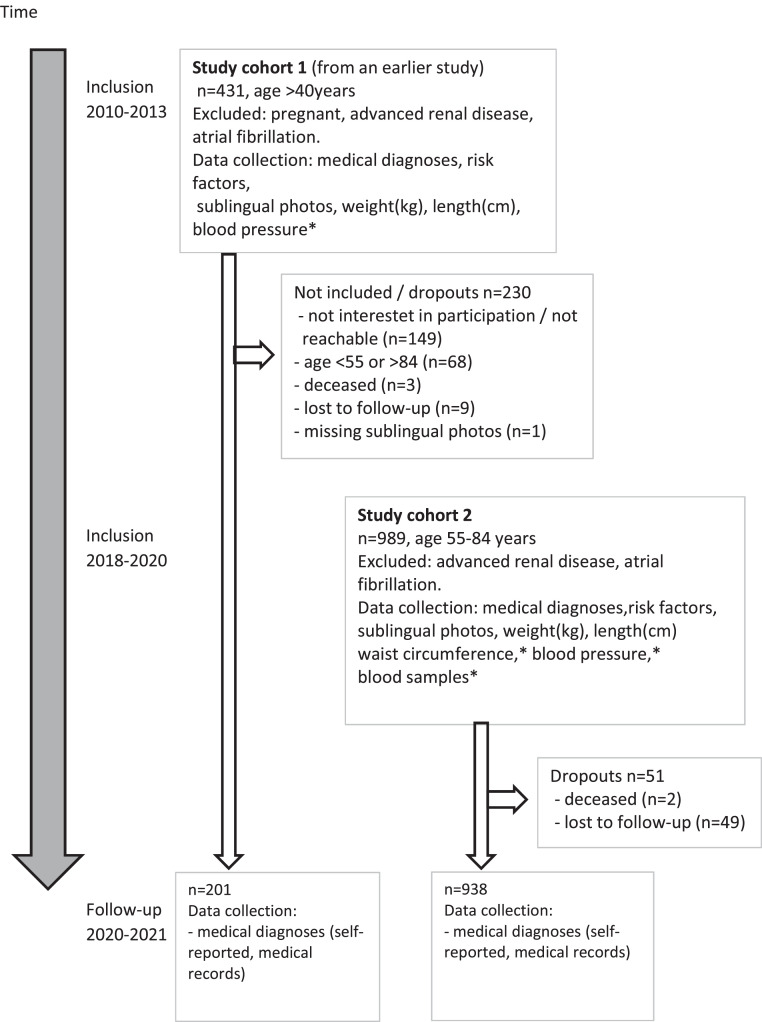
Flowchart of the study. Cohort 1 was included in 2010–2013 (n = 431) and cohort 2 was included in 2018–2020 (n = 989) and followed up in 2020–2021. Cohort 1 was included in 2010–2013 for another study with the aim of studying a possible connection between sublingual varices and hypertension. The blood pressure devices used were not validated for pregnancy, advanced kidney disease, or atrial fibrillation; therefore, they were excluded. In the study cohort 2 that was included in 2018–2020, a relationships between SV and risk factors for CVD have also been studied (high blood pressure, high blood sugar, dyslipidemia, abdominal obesity). The same blood pressure devices were used in this study; hence, the same exclusion criteria were also used. *Data not used in this study.

This prospective observational study was designed with the goal of incorporating a large study population and a lengthy observation period. The study drew participants from a prior investigation^5^ and a new cohort. The first cohort was enrolled between 2010 and 2013 (age >40 years, n = 431), and the subsequent one was recruited from 2018 to 2020 (age 55–84 years, n = 989) (Figure 1). All study participants were consecutive visitors for an annual dental checkup. The patients were from 2 dental clinics (a municipal clinic and a private clinic with a total of 13,000 listed patients) in a small Swedish town. Exclusion criteria included atrial fibrillation, advanced renal disease requiring dialysis, and pregnancy, due to the inapplicability of the blood pressure devices for these groups (Figure 1).

### Ethical approval

This study was approved by the Regional Research Ethics Committee at the University of Lund (EPN 2009/204, 215/294, 2018/60) and was conducted in accordance with the Helsinki Declaration. Informed consent was obtained from all individual participants included in the study.

### Data collection

All participants received both written and verbal information about the study, after which they signed an informed consent form. In connection with the inclusion in the study, information about background, smoking, and medical conditions was collected via a questionnaire. The sublingual veins were documented with photos, one on each side, and measurements of height (cm) and weight (kg) without shoes were registered (the study is earlier described).^9^ As a follow-up (between September 2020 and November 2021), the participants answered a questionnaire about medical conditions either by phone or by post. Diagnoses were collected according to the ICD-10 from the medical records of the participants at the medical clinic at the nearby hospital.

### Data processing

Age was registered in whole years. The questions about smoking were formulated as “Do you smoke?” with the following answers: Yes, daily; Yes, sometimes; No. These answers were dichotomised to Yes (daily/sometimes)/No. The question about diseases was formulated as follows: “Do you have any of these diseases diagnosed by a physician?” with the following answers: Yes; No; I don´t know. The answers were dichotomised to Yes/No, where “I don´t know” was counted as “No.” Body mass index (BMI) was calculated as body weight (kg) divided by squared height (m²). The main outcome variables were new IHD and new cerebrovascular disease. The participant answered the following questions: Have you been diagnosed by a physician with a myocardial infarction? (Yes/No/I don´t know), and approximately which year. The same formulation and answer was asked about angina, stroke/transient ischaemic attack (TIA), diabetes mellitus type 2 (DM2), hypertension, and high blood lipid levels. The responses were dichotomised into Yes and No, where “I don't know” was included in No. All positive and negative answers concerning a heart disease and stroke/TIA were validated by the medical records at the nearby hospital. The variables myocardial infarction and angina were merged into a new variable “IHD.”

A new IHD was defined as presence of a diagnosis (myocardial infarction, angina pectoris) at follow-up not reported by the participant at baseline or a newly registered diagnosis (I200–219, I248–249) in their medical records dated after inclusion in the study.

A prior IHD was defined as presence of a diagnosis (myocardial infarction, angina pectoris) at baseline reported by participants or a registered diagnosis (I200–219, I248–249, I250–259) in their medical records dated before inclusion in the study.

A new cerebrovascular disease was defined as presence of a diagnosis (stroke/TIA) at follow-up not reported by the participant at baseline or a newly registered diagnosis (I600–649, G458–459, Z867C) in their medical records dated after inclusion in the study.

A prior cerebrovascular disease was defined as presence of a diagnosis (stroke/TIA) at baseline reported by participants or a registered diagnosis (I600–649, I678, G458–459, I690–698, Z867C) in their medical records dated before inclusion in the study.

**Fig. 2:**
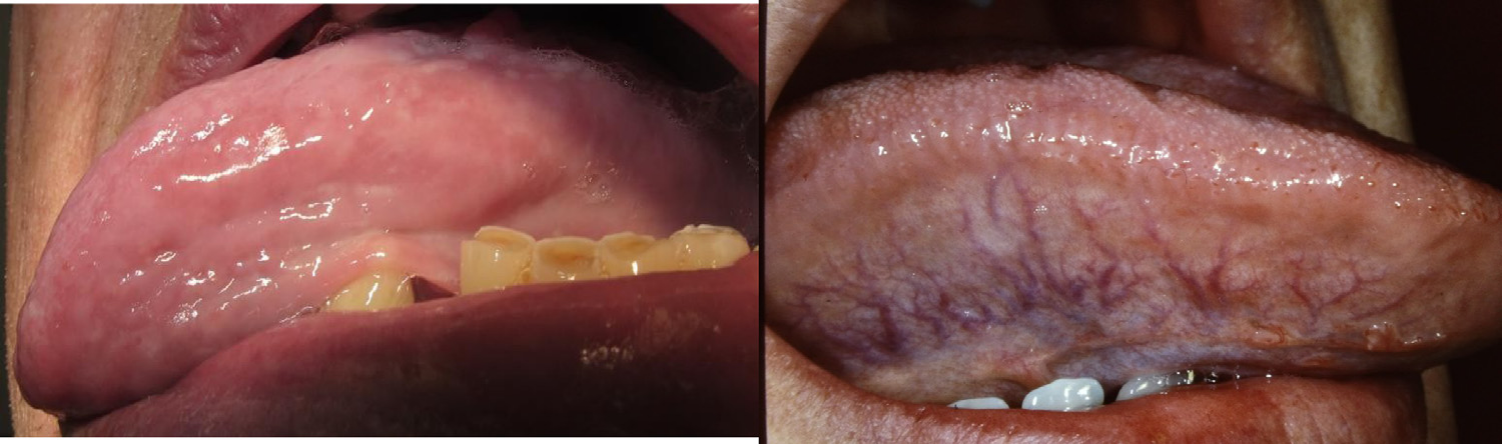
None/few sublingual varices (grade 0) (left) and moderate/severe sublingual varices (grade 1) (right). Written consent obtained from the patients.

The sublingual veins were classified as none/few visible SV (nSV) or medium/severe SV from the digital photos of the left and right underside of the tongue (Figure 2). To ensure reliable classification, 2 dentists were trained to evaluate around 50 digital photos under the guidance of one of the authors (LH). The classification was performed by 2 experienced dentists blinded to each other's assessment and the participant's medical data, in accordance with the method used in a previous study.^5^ Consensus was reached in cases where the assessment differed between observers.

### Statistical analysis

Based on their sublingual veins at baseline, the study population was divided into 2 subgroups: nSV and SV. Chi-squared tests (for ordinal data) and Student *t* test (for continuous data) were used for group comparisons regarding background variables, smoking habits, BMI, and diagnoses. The association of the independent variables (IHD and cerebrovascular disease) with SV was analysed using multiple logistic regression (the enter method) where the estimated values were adjusted for sex, age, and smoking. In the models, sex (male = 0, female = 1), age (q^1^ ≤60 years, q^2^ = 61−66, q^3^ = 67−72, q^4^ ≥ 73) and smoking (no = 0, yes = 1) were analysed as categorical variables. Odds ratio (OR) was calculated with 95% confidence interval (CI). Multicollinearity was assessed using multiple linear regression analysis, with the tolerance limit set at variance inflation factor = 3.0. The agreement between the raters of the sublingual images was measured using Cohen kappa (the interrater reliability). All tests were 2-tailed, and statistical significance was set at *P* < .05. All analyses were conducted using SPSS 27.0.

## Results

The initial study group encompassed 1420 participants, of whom 281 were lost due to various reasons: 149 could not be reached or declined participation, 68 were disqualified on account of inappropriate age, 58 did not engage in follow-up, 5 were deceased, and 1 was excluded due to absent sublingual photographs (Figure 1). The study population (n = 1139) had a mean age of 66 years and consisted of 54.3% women (Table 1). At the start of the study, 32.9% stated a diagnosis of hypertension, 5.2% DM2, and 19% dyslipidaemia. Of the participants, 377 (33%) were classified as having SV. The group with SV had a higher average age and BMI and consisted of more men and smokers. Those with SV had higher blood pressure levels and more DM2 and dyslipidaemia than those with nSV (Table 1).

**Table 1:** Background variables at baseline for the study cohort.

	Full cohort (n= 1139)	nSV (n = 762)	SV (n = 377)	*P* value	CI
Sex (n = 1139), % (male/female)	45.7/54.3	42.3/57.7	52.5/47.5	.001*	
Mean age (n = 1139), y (SD)	66.4 (7.3)	65.2 (7.0)	68.7 (7.4)	<.001*	−4.38 to −2.62
Smoker, % (No.)	10.0 (114)	8.1 (62)	13.8 (52)	.003*	
Hypertension, % (No.)	32.9 (374)	27.1 (206)	44.7 (168)	<.001*	
DM2, % (No.)	5.2 (59)	3.7 (28)	8.3 (31)	.001*	
Dyslipidaemia, % (No.)	19.0 (214)	16.4 (123)	24.3 (91)	.001*	
BMI (n = 1117), kg/m^2^ (SD)	26.2 (4.2)	26.0 (4.2)	26.7 (4.1)	.007*	−1.23 to −0.19
Lower limb varices, % (No.)	18.5 (210)	18.1 (138)	19.1 (72)	.678	

A comparison between those with no sublingual varices (nSV) and those with sublingual varices (SV) is presented with *P* values and 95% confidence intervals (CIs).

DM2, diabetes mellitus type 2; BMI, body mass index; MI, myocardial infarction.

⁎ Statistically significant.

### IHD

Of the participants, 69/1139 (6.0%) had an IHD. IHD was more frequent amongst those with SV 38/377 (10.1%) than amongst those with nSV 31/762 (4.1%) (*P* < .001) (Table 2). After dividing the IHD into preexisting or newly diagnosed (before or after entering the study), the latter were found to be more prevalent amongst the SV group: 17 out of 377 (4.5%) compared to the nSV group: 14 out of 762 (1.8%) (*P* = .009).The association between SV and new IHD remained after adjustment for sex, age group, and smoking (OR, 2.26; 96% CI, 1.07—4.76) (Table 3).

**Table 2:** Prior and new IHD and prior and new stroke/TIA during the study period in the whole study population and in the subgroup with nSV and with SV.

	Full cohort (n = 1139)	nSV (n = 762)	SV (n = 377)	*P* value
Prior IHD, No. (%)	69 (6.0)	31 (4.1)	38 (10.1)	<.001*
New IHD, No. (%)	31 (2.7)	14 (1.8)	17 (4.5)	.009*
Prior stroke/TIA, No. (%)	66 (5.8)	37 (4.9)	29 (7.7)	.054
New stroke/TIA, No. (%)	31 (2.7)	15 (2.0)	16 (4.2)	.026*

A comparison of events is made between no sublingual varices (nSV) and sublingual varices (SV) and is presented with *P* values.

IHD, ischaemic heart disease; TIA, transient ischaemic attack.

⁎ Statistically significant.

**Table 3:** The association between sublingual varices and the independent variables new events of IHD and new events of stroke/TIA by means of logistic regression analysis controlled for age (categorical), sex, and smoking.

	OR	*P*	CI
New IHD (n = 1139)	2.26	.033*	1.07–4.76
- Sex	0.63	<.0001*	0.48–0.81
- Age	1.57	<.0001*	1.39–1.77
- Smoking	2.08	<.0001*	1.38–3.12
New stroke/TIA (n = 1139)	1.77	.132	0.84–3.71
- Sex	0.62	<.0001*	0.48–0.81
- Age	1.56	<.0001*	1.39–1.76
- Smoking	2.01	.001*	1.37–3.09

The results are expressed as *P* values and 95% confidence intervals (CIs).

IHD, ischaemic heart disease; TIA, transient ischaemic attack.

⁎ Statistically significant.

### Cerebrovascular disease

Of the participants, 66 (5.8%) reported having had a stroke/TIA (Table 2). These were unevenly distributed between those with SV (29/377; 7.7%) and those with nSV (37/762; 4.9%) (*P* = .054). Similar to IHD, when stratified into preexisting or newly occurring strokes/TIAs (before or after the study onset), the latter were more common in the SV group: 16 out of 377 (4.2%), than in the nSV group: 15 out of 762 (2.0%) (*P* = .026). The association between SV and new stroke/TIA did not remain in the multivariate analysis after adjustment for sex, age group, and smoking (OR, 1.77; 95% CI, 0.84—3.71) (Table 3). The Cohen kappa coefficient was 0.59.

## Discussion

The results from this study show a novel association between SV and both newly diagnosed and preexisting IHD but not with newly occurring stroke/TIA events. These are new findings never shown or studied before, and therefore there are no other studies to compare the results with.

However, there are indications from previous cross-sectional studies of a possible association between SV and CVD with hypertension included in the CVD variable.^4^^,^^6^^,^^12^ In a study where hypertension was not included in the CVD variable, no relationship was found between SV and CVD; in this study, the study population was younger, with a mean age of 39 years.^7^ Other studies have found associations between SV and self-reported risk factors for CVD: hypertension, DM2,^2^^,^^7^ dyslipidaemia,^10^ and smoking.^2^^,^^4^, ^5^, ^6^^,^^9^^,^^13^ Some studies have found a relationship between a measured high blood pressure and SV (OR > 2).^5^^,^^13^ In one study, blood samples were taken from the study population, and a connection was found between SV and low high-density lipoprotein cholesterol and with high b-glucose (OR = 1.8) after adjustment for sex and age.^9^ In the mentioned study, a relationship between measured abdominal obesity and SV (OR = 1.7) adjusted for sex and age was also demonstrated.^9^

In this study, we found a connection between SV and new events of IHD consistent with the previous connections with cardiovascular risk factors. This finding indicates that there may be a temporal relation wherein SV develops before IHD. However, due to the pioneering nature of these findings and the limited sample size (only 31 events of IHD and stroke/TIA, respectively), we advise caution in interpreting these results until further validation studies are conducted.

Several studies have suggested an association between sublingual varices and hypertension, hinting at concurrent impacts on the venous and arterial systems.^5^^,^^7^^,^^10^

Matrix metalloproteinases (MMPs) are a group of endopeptidases involved in vascular tissue remodelling.^18^^,^^19^ They may influence both arterial and venous remodelling and are involved in various diseases such as atherosclerosis, hypertension, and lower extremity venous disease.^19^ An illness process affecting MMPs could instigate changes in both arteries and veins, providing a pathophysiologic link between these 2 vascular systems. If our findings are validated in future studies, this could indicate the presence of a pathophysiologic mechanism impacting both the arterial and venous systems. Moreover, establishing a link between SV and IHD could hold clinical significance in fostering improved collaboration between dental and health care practitioners and in early identification of individuals at risk for IHD.

### Methodologic considerations

The study sample comprised 2 cohorts included during different time periods. The initial cohort was included from 2010–2013, with the aim of investigating the relationship between SV and hypertension. The second cohort was added from 2018-2020. Data collection for both cohorts was executed in a similar manner. Our original objective was to involve 300 individuals from the first cohort and 1000 from the second. However, the COVID-19 pandemic impacted participant recruitment and follow-up, causing us to fall short of our recruitment goal. The extended follow-up period inadvertently lengthened our observation period, leading to an increased number of end points (new IHD and new stroke/TIA), partially offsetting the smaller participant number. This deviation is acknowledged as a limitation of our study.

The inclusion was similar for both periods. It was a consecutive selection of patients attending their ordinary dental clinic for a yearly checkup. The population included both healthy individuals and those with disease at different ages. In Sweden, dental care is subsidised by the state, which means that a large proportion of the population regularly goes to the dentist: 77% over a 3-year period.^14^ For this reason, the study population can be considered to be close to a representative sample of the population. The part of the study population that was included in 2010–2013 is included in previously published studies,^5^^,^^9^ and the part that was included in 2018–2020 is included in the study from 2022.^9^ The classification of sublingual veins as SV or nSV presents another limitation, due to its subjective nature. To mitigate the impact of this subjectivity, we performed a validation process involving 2 dentists who classified all the photographs. Recently, efforts have been made to devise more objective methods for classifying digital photos.^15^^,^^16^ The classification process was blinded and resulted in a Cohen kappa coefficient of 0.59.

As SV are associated with high age, some individuals experience SV when they get older. The stability of the sublingual vascular status over an 8-year period is 77%; 81% of those with nSV were unchanged whilst 64% of those with SV still had them after 8 years.^17^ The transformation process of the sublingual vessels from nSV to SV and vice versa is likely to occur gradually.

The outcome variables new events of IHD and new events of cerebrovascular disease were gathered prospectively based on a self-reported questionnaire. The questionnaire was not validated, but the questions were formulated in a simple manner with a limited number of possible answers. Diagnoses were also collected from the medical records of participants at the medical clinic at the nearby hospital. Some diagnoses not reported by the participants were found in the medical record, which was probably explained by recall bias. Some participants reported that they had had an event of IHD or stroke/TIA which could not be verified in the medical record at the nearby hospital. These events were probably recorded in another hospital record (which we did not have access to), and they were included.

However, a limitation to consider is that we only included a limited number of covariates (gender, age, smoking) in our model. It is possible that there may be additional confounding factors influencing the relationship between SV and IHD that were not accounted for in our analysis. As such, the results should be interpreted within the context of these outlined strengths and limitations, and we do not believe that they significantly impact the conclusions drawn from our findings.

## Conclusions

We found an association between SV and new events of IHD with a temporal relationship where SV were detected before the events of IHD. This novel finding suggests a possible shared pathophysiology for IHD and SV. Given the ease of visualising sublingual vessels, these findings could have significant implications for identifying individuals at risk of IHD.

## Conflict of Interest

None disclosed.
